# From Control to Care: Reframing Medication Access Policies Through Public Health Ethics

**DOI:** 10.1017/jme.2025.10220

**Published:** 2026

**Authors:** Kaitlyn Jaffe, Elizabeth A. Evans

**Affiliations:** Health Promotion and Policy, University of Massachusetts Amherst, United States

**Keywords:** medication access, medications for opioid use disorder, carceral health systems, public health ethics

## Abstract

Dantuluri examines the ongoing prescription stimulant shortage driven by the Drug Enforcement Administration’s quota restrictions and an ineffective and fragmented system of drug governance. We extend this analysis to carceral health systems, which operate under similar logics of control in managing risks, diversion, and liability around medications for opioid use disorder. In drawing these parallels, we explore how perceptions of risk, suspicion, and restrictive oversight can produce scarcity, reinforce stigma, and elicit judgments around “deservingness” that may ultimately widen treatment gaps. We conclude with actionable recommendations that align with public health ethics to promote equitable access to evidence-based treatment.

In her article, Dantuluri examines the ongoing prescription stimulant shortage, which she argues stems from a “confluence of regulatory and systemic failures,” with significant impacts on patients with attention-deficit/hyperactivity disorder (ADHD).^1^ She traces the shortage to the Drug Enforcement Administration’s (DEA’s) restrictive quota system for Schedule II stimulants that caps production. Dantuluri argues the quotas have not kept pace with the rise in ADHD diagnoses and prescriptions, an increase partly attributed to the expansion of telehealth access during the COVID-19 pandemic. These federal restrictions, along with state limits on prescription transfers and refills, create significant barriers to medication continuity, especially for low-income and rural patients. Dantuluri also describes an inefficient and fragmented system of drug governance in the United States, where multiple health, drug, and social service agencies have overlapping roles and contradictory mandates around regulating prescription medications. While some agencies have developed piecemeal measures to address the shortage, Dantuluri argues “adaptive, data-driven reform” is needed to improve medication access.

Building on Dantuluri’s analysis, we expand the discussion to system-level impacts, focusing on our areas of expertise around health equity and substance use within carceral health systems. We see parallels between the DEA restrictions outlined by Dantuluri and carceral system practices that both are shaped by logics of control, or the institutional mechanisms that prioritize managing potential risks over rapid uptake of innovations in evidence-based health care.^2^

The DEA’s quota system and carceral health care systems are both oriented toward managing institutional risk and preventing medication diversion. Dantuluri highlights how the DEA controls stimulant availability through drug scheduling, production quotas, and system oversight of distribution. In carceral settings, medications with any potential for abuse are also tightly controlled and surveilled to manage diversion, security, and liability risks.^3^ Some jurisdictions limit dispensing certain evidence-based treatments in carceral settings (e.g., medications for opioid use disorder, ADHD) for fear of diversion, reflecting extreme risk avoidance at the expense of patient well-being.^4^ While concerns around misuse and diversion are valid, they must be addressed through effective strategies that prioritize health (e.g., provider education, patient-centered substance use treatment, harm reduction approaches), rather than withholding health care or using other punitive tactics that deepen health disparities.^5^

Moreover, these punitive oversight mechanisms can reflect underlying biases and suspicion around legitimate prescription use. Multilevel surveillance tools have been created to monitor, document, and penalize both patients and providers suspected of misuse (e.g., prior authorization requirements, electronic health record monitoring, Prescription Drug Monitoring Databases, etc.).^6^ In carceral settings, oversight protocols are embedded within the medication dispensing process. For instance, incarcerated people who receive medications for opioid use disorder are typically subject to more intensive scrutiny (e.g., mouth checks, escort by correctional officers, random urine tests, housing unit inspections, video surveillance) and/or penalized for real or perceived lack of compliance with dispensing protocols.^7^ While these practices may be necessary for safety, they can also propel institutional distrust and undermine patient-centered care.

Relatedly, restrictions on medication production and access produce health care scarcity. When medications are limited, the allocation of these rationed resources often depends on subjective evaluations of who “deserves” treatment (e.g., responsible, compliant patients), likely widening gaps in care.^8^ For example, some jails induct individuals onto medications for opioid use disorder upon entry only if they were engaged in community-based treatment immediately prior to incarceration, while other jails delay initiating medication until just prior to release.^9^ This practice creates two groups within jails: those who continue receiving treatment immediately upon incarceration, and those who go without treatment despite their medical need. Such allocation disadvantages those who could benefit most — people untreated in the community who enter jail — and represents missed opportunities for health care.

These perceptions of risk, suspicion, and scarcity have consequences. First, medication scarcity produces treatment gaps in community and carceral settings, forcing patients to taper from medications or pursue less effective alternatives. The Iron Law of Prohibition and lessons from the opioid crisis demonstrate that supply-side restrictions can shift demand to illicit markets, increasing drug-related harms.^10^ In carceral settings, the rationing of medication, along with housing treated and untreated people together, creates avoidable conditions for medication diversion, which can prompt punitive policies that further destabilize care.^11^ Second, perceptions of health care scarcity contribute to cultural attitudes around deservingness and entrench stigma around drug use.^12^ This drug- and treatment-related stigma can deter patients from accessing legitimate pharmacotherapies,^13^ and, in carceral contexts, raises post-release mortality risk.^14^ Finally, restrictions on drug scheduling and production can limit opportunity for research and innovation. Despite the current polysubstance overdose crisis and substantial burden of problematic stimulant use (e.g., cocaine, methamphetamine, prescription stimulants) in the US,^15^ there are no federally approved medications to treat stimulant use disorder.^16^ While international trials show promising evidence for treatment using prescribed stimulants, US researchers face constraints on testing and development related to these regulatory barriers.^17^

Dantuluri poses a question: “What is the most effective way to balance concerns about stimulant misuse while ensuring equitable access to necessary treatments?” We propose approaching this issue by drawing on public health ethics, specifying that public health authorities have an obligation to communicate with and involve constituent communities, along with experts, to identify health threats and weigh the risks and benefits of responses.^18^ Medication scarcity, as highlighted by Dantuluri, disproportionately harms low-income and rural populations, and, we would add, criminalized populations. Because marginalized populations often have limited influence over health policies, a public health approach calls for amplifying the voices of affected communities and prioritizing them for access to essential health services, rather than excluding them from care.

We recommend actionable commitments consistent with these ethics:

- Ensure swift access to evidence-based medications to treat opioid use disorder in carceral settings, including at intake, during incarceration, and upon release.
- Build on, rather than roll back, pandemic-era progress in telehealth, outreach models that increase medication for opioid use disorder uptake, and harm reduction practices.
- Address medication restrictions through participatory governance, transparency, and alignment with the objectives of public health.

Taken together, these commitments align with public health obligations to prevent harm and promote equitable access to care.
